# Barriers and facilitators to implementing the European code of cancer practice in a rural and coastal setting: a qualitative study

**DOI:** 10.1007/s10552-026-02225-1

**Published:** 2026-08-08

**Authors:** Shanice Thomas, Hannah Cumberland Keeble, Erica Brown, Sarah R. Baker, Samuel Cooke, Mark Lawler, Kathie McPeake, Kathy Oliver, Peter Selby, David Nelson

**Affiliations:** 1 Lincoln Institute for Rural and Coastal Health, University of Lincoln, Brayford Pool, Lincoln, LN6 7TS UK; 2 Lincoln Medical School, Universities of Nottingham and Lincoln, Lincoln, UK; 3 NHS Lincolnshire Integrated Care Board, Sleaford, UK; 4 Macmillan Cancer Support, London, UK; 5 Faculty of Medicine and Health, University of Leeds, Leeds, UK; 6 International Brain Tumour Alliance, Surrey, UK; 7 Johnston Cancer Research Centre, Queen’s University Belfast, Belfast, UK

**Keywords:** Cancer care, European code of cancer practice, Rural, Coastal, Qualitative, Survivorship

## Abstract

**Purpose:**

The European Code of Cancer Practice (ECCP) is a patient-centred framework for good clinical cancer practice, to improve outcomes for all of Europe’s patients [1]. Given challenges with applications to rural regions, the study aimed to explore the barriers and facilitators to implementing the ECCP in a rural, coastal, and urban context.

**Methods:**

We conducted semi-structured individual and group interviews with nine acute cancer professionals; nine non-clinical professionals; and four people with lived experience of cancer. Interviews were analysed using reflexive thematic analysis, underpinned by a constructivist approach.

**Results:**

Three themes were developed across all groups. 1) ‘Fragmented communication pathways limits access’ to information-sharing between services, and about support and research, linked to workforce challenges. 2) ‘Geographic and infrastructural disparities between urban, rural, and coastal areas’ highlight barriers to accessing and delivering care, including transport issues, alongside place-specific facilitators to wellbeing. 3) ‘Unmet needs for living well during and after treatment’ denotes financial, work-related, and psychological challenges through treatment and survivorship. These barriers transcended across all ECCP domains.

**Conclusion:**

Workforce challenges that limit information-sharing about support and research, and gaps in post-treatment support, must be addressed to meet the ECCP aim to ensure equitable cancer care across geographical locations.

**Supplementary Information:**

The online version contains supplementary material available at https://doi.org/10.1007/s10552-026-02225-1.

## Introduction

**Fig. 1:**
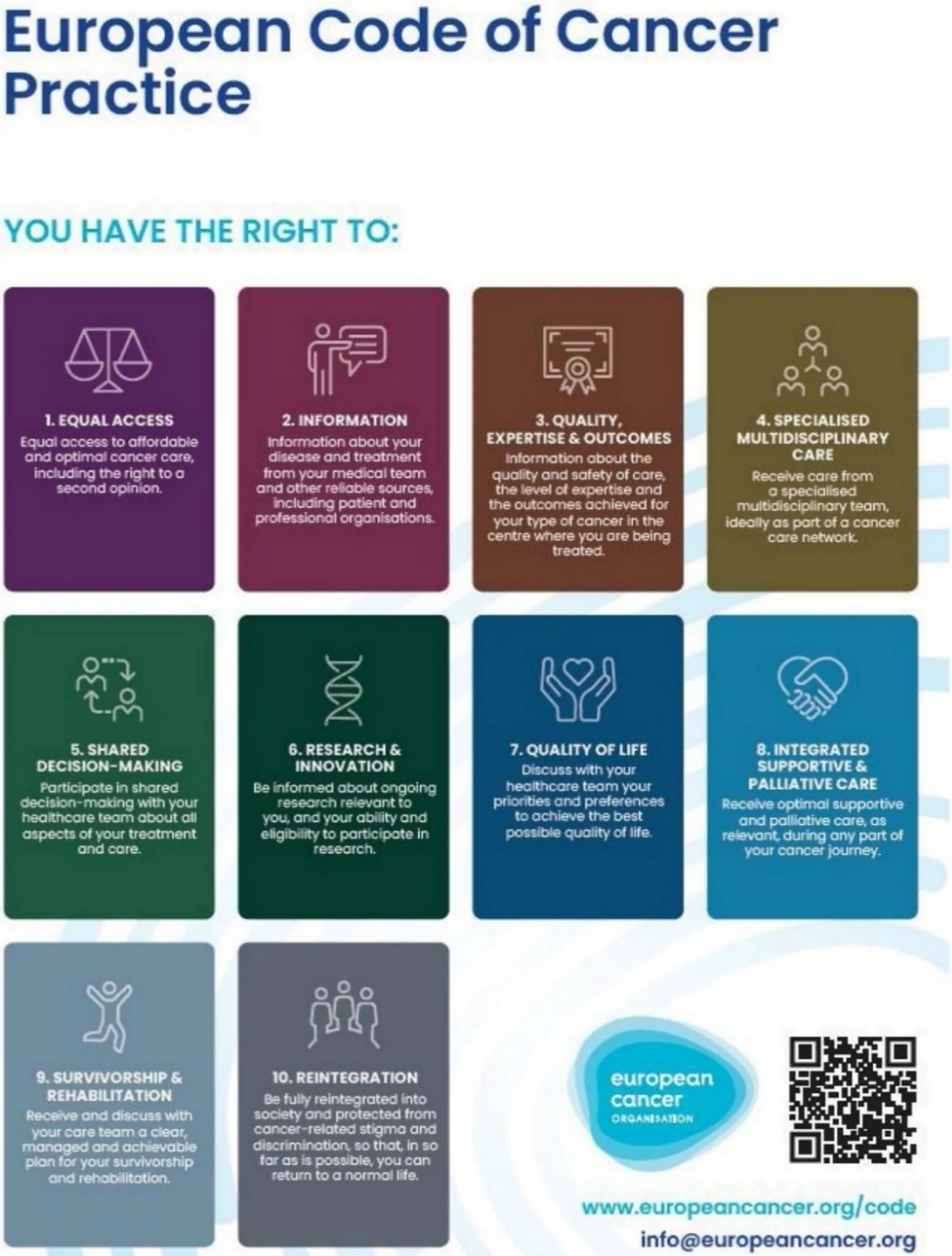
Ten key patient rights outlined by the ECCP, taken from Lawler et al (2021) [13]. Figure used with permission from the European Cancer Organisation on 4 June 2026

With advances in prevention, detection, and treatment, many cancers are increasingly managed as a chronic condition, leading to a growing population of long-term survivors [2]. This success brings new challenges, as survivors often face a range of physical and psychological co-morbidities, increased comorbidities with ageing which require ongoing, complex care [3, 4] and other challenges aligned to the wider determinants of health [5]. However, the quality and accessibility of this care vary significantly, particularly within rural and coastal communities where people face greater financial, travel, and other access-related barriers to cancer care [6–8], creating disparities in patient outcomes. To address these disparities, the European Code of Cancer Practice (ECCP) was developed by the European Cancer Organisation to empower patients and guide healthcare systems by establishing ten rights [9], outlined in Fig. 1.

Building on the European Cancer Patient Bill of Rights, the ECCP aims to ensure that all patients receive the best available care throughout the cancer care pathway, regardless of their location or socioeconomic status [10]. As an evidence-based, patient-centred framework, it was co-produced with cancer patients, patient advocates, and cancer professionals and serves as an educational tool to assist citizens throughout their care journey, from diagnosis to survivorship (including supportive and palliative care) [1]. Despite its endorsement, including by the European Commission [9], implementation of the ECCP remains challenging in practice, particularly within rural, coastal, and remote regions, where pre-existing health inequalities are often more pronounced [11]. A recent survey of oncology professionals in Poland found that some EECP rights were particularly challenging and less feasible to implement, due to workforce and attitudinal barriers [12]. For example, equal access to optimal care and survivorship and rehabilitation planning. Yet, evidence on barriers and facilitators to implementing the ECCP in the United Kingdom (UK) and other European contexts remains limited. It is therefore difficult to robustly evaluate current service delivery against these patient-centred rights, or to assess the utility and impact of the ECCP for services [11, 13].

Addressing these implementation gaps is crucial to support the goal of the ECCP to improve patient cancer care globally, which must include, not leaving behind, people affected by cancer in rural and coastal communities. This is particularly important as the number of people living with cancer and multi-morbidities rises, while healthcare systems across Europe face ongoing resource constraints that affect care delivery [4]. Whilst the ECCP was co-produced with people with lived experience and healthcare professionals, it is unclear how these groups each perceive barriers and facilitators to implementation in practice. This study addresses this evidence gap by examining the implementation and impact of this policy code in the experience and delivery of cancer care, focusing on rural, coastal, and urban settings.

Lincolnshire, the second-largest county in England, where over 35,000 people are living with cancer [5], exemplifies the above health and healthcare challenges. It is a geographically-diverse region with a significant rural and coastal population, areas of high deprivation, an ageing demographic, and limited transport infrastructure [5]. Cancer survival outcomes in remote areas are often poorer than in urban areas, linked to complex factors including reduced screening participation, socioeconomic disadvantage, and greater travel distances to access specialised services [14]. While the challenges of rural and coastal cancer care are widely documented [15–17], the specific barriers and facilitators to implementing the ECCP framework within such contexts is critically underexplored. This study addresses this evidence gap by examining the implementation and impact of this policy code in the experience and delivery of cancer care, focusing on rural, coastal, and urban settings. The study aimed to answer the following research questions, using the definitions in Table 1:

1. What are the barriers to implementing the ECCP rights regarding the delivery of cancer care in rural, coastal, and urban settings?
2. What are the facilitators that promote implementing the ECCP rights in the delivery of cancer care in rural, coastal, and urban settings?

**Table 1:** ECCP rights and definitions [1], used in this study

ECCP rights	Definition
1. Equal access	Patients should have equal access to affordable and optimal cancer care, including the right to a second opinion
2. Information	Patients should receive information about their disease and treatment from your medical team and other reliable sources, including patient and professional organisations
3. Quality, expertise and outcomes	Patients should receive information about the quality and safety of care, level of expertise and the outcomes achieved for their type of cancer in the centre where they are being treated
4. Specialised multidisciplinary care	Patients should receive care from a specialised multidisciplinary team, ideally as part of a cancer care network
5. Shared decision-making	Patients should participate in Shared Decision-Making with their healthcare team about all aspects of their treatment and care
6. Research and Innovation	Patients should be informed about any ongoing research and innovation within the service delivering their care
7. Quality of Life	Patients should expect to live as normally as possible with optimum QoL following diagnosis, during treatment and through survivorship
8. Integrated supportive and palliative care	Patients should have access to supportive and palliative care at any point, from diagnosis, as survivors or in end-of-life care
9. Survivorship and rehabilitation	Patients’ care should be supported in their needs as a cancer survivor
10. Reintegration	Patients should be able to reintegrate into society to the fullest extent possible

## Methods.

### Conceptual framework

This study used the ECCP [1] as a conceptual framework for exploring professional and patient perspectives on cancer care in rural, coastal, and urban settings. The ten ECCP rights informed the data collection materials and analytic process, outlined below. More broadly, our approach was informed by Levesque’s Conceptual Framework of Access to Health [18], which sees access to healthcare as multi-faceted and facilitated by health system factors (such as approachability, acceptability, and, affordability) and patient-level factors (such as individuals’ ability to perceive need and reach services). This framework informed our recognition of both professional and patient perspectives, and has been used to examine other health-related inequalities in high-income rural settings [19].

### Study design

A qualitative design was employed, using semi-structured interviews to gather in-depth perspectives on cancer care against the ECCP rights, in Lincolnshire (East Midlands, England, UK). The study used a constructivist ontological paradigm, appreciating that patient and professional cancer care experiences are subjective and will be shaped by individuals’ social contexts [20]. Ethical approval was granted by the University of Nottingham Faculty of Medicine and Health Sciences Research Ethics Committee (reference: FMHS 279–0924).

### Operationalising geographic setting

As this study examines professional and patient subjective perspectives on rural and coastal cancer care, we did not quantitatively measure geographic settings. We appreciate heterogeneity between rural, urban, and coastal settings and narrowly defining them could simplify complex, context-specific realities [21]. We define rural and urban settings broadly in relation to the Office for National Statistics 2021 Rural Urban Classification [22], widely used in research, which considers density, settlement forms, population size, and access to towns and cities. Whilst there is no universal definition of ‘coastal’ [23], recent work has conceptualised coastal cancer care as the ‘design, delivery and evaluation of research, policy, programmes and services addressing the needs and challenges of communities residing near the coast’, such as within 10 km in the UK and contextual adjustment for other countries [21]. This recognises that cancer needs and challenges in coastal areas are shaped by place-specific socioeconomic, environmental, and system factors [21]. It encompasses the full cancer care continuum from prevention, diagnosis, treatment, to survivorship, palliative and end of life care, including patient, carer, and workforce perspectives.

### Participants and recruitment

A purposive sampling strategy was employed to recruit participants with diverse experiences relevant to the research questions. The sample consisted of nine acute clinical cancer healthcare providers, nine non-clinical cancer care specialists, and four individuals with lived experience of cancer (in remission), including a public representative who was also an informal caregiver. Participants were recruited through established professional networks and the Macmillan Living with Cancer Programme. Interested individuals self-selected by contacting the research team after receiving an email invitation and participant information sheet. All participants provided written informed consent.

### Data collection

Data were collected between October-December 2024 in four group interviews and ten individual interviews using a semi-structured approach, conducted by EB and HCK. DN and SC observed initial interviews (with participants’ consent) to advise on process but did not intervene. Interviews (duration: 16–73 min; mean: 47 min) were held online via Microsoft Teams or face-to-face at the University of Lincoln, based on participant preferences. Each participant was sent the ECCP before the interview, and the research team developed an interview guide to prompt discussion on the challenges, opportunities, and differences in implementing the ECCP across rural, coastal, and urban Lincolnshire (see supplementary materials). Participants were not known to EB or HCK before data collection. Questions focused primarily on the latter five ECCP rights to identify short-medium term interventions in these areas, while a semi-structured approach allowed space for participants to reflect on all ECCP rights. All interviews were audio-recorded and transcribed verbatim using an automated function, followed by manual anonymisation. Demographic data, beyond working or living in rural or coastal Lincolnshire, was outside the scope of this study.

### Data analysis

The transcripts were analysed using reflexive thematic analysis (RTA), following the six-phase process outlined by Braun and Clarke [24], which recognises researchers’ active roles in co-constructing knowledge with participants. EB and HCK undertook ‘data familiarisation’ (phase 1) by reading the transcripts multiple times and noting analytic insights. EB and HCK independently coded the transcripts (phase 2) to generate concise, descriptive labels for specific data segments [24]. RTA allowed for a hybrid approach to coding; the first phase of coding was inductive, to identify insights about that were grounded more directly in the data and according to what mattered to participants, with distance from the pre-determined ECCP domains. The second phase of coding deductively focused on the ECCP domains, yet there were many synergies between the coding phases as the interview topic guide was structured using the ECCP. These codes represented EB’s and HCK’s interpretations of patterns of meaning in the data. EB and HCK then compared codes, which enhanced reliability [25].

To generate initial themes (phase 3), EB and HCK individually grouped codes according to concepts and features which they considered represented broader, patterned units of meaning [25]. They developed and reviewed the viability and distinctiveness of each initial theme in phase 4 [24], then each defined and named their themes in phase 5, whilst iteratively returning to the data. EB and HCK then wrote up thematic narratives (phase 6), which evolved into a collaborative process as DN, SB, and ST amalgamated and refined these narratives, whilst staying close to the original codes and transcripts, to articulate the final themes. NVivo 14 software was used to systematically manage the data.

### Reflexivity and rigour

To ensure rigor and transferability, this study aligns with the consolidated criteria for reporting qualitative research checklist to report the study processes in detail (see supplementary materials) [26], and RTA quality standards [27]. Regular discussions with DN and SC aided reflexive dialogue for EB and HCK to validate their developing interpretations, codes, and themes, which supported analytical rigour and credibility. Dependability of this study was enabled using a semi-structured interview guide, which supported consistent data collection.

## Results

The analysis identified three themes that encapsulate the barriers and facilitators to implementing the ECCP from the perspectives of non-clinical cancer care specialists (n=9), acute cancer healthcare providers (n=9), and people with lived experience (n=4), including an informal caregiver. These participants, and their distribution across individual and group interviews, are outlined in Table 2. The first theme examines ‘Fragmented communication pathways’ that limited access to information about support and research. The second theme, ‘Geographic and infrastructural disparities between urban, rural, and coastal areas’, identifies variable experiences of accessing and delivering cancer care across Lincolnshire. The third theme, ‘Unmet needs for living well during and after treatment’, identifies barriers in current support. Each theme is evidenced with indicative codes and quotes in Table 3 and the narrative below.

**Table 2:** Total number of participants by group and role

Group (n)	Participant role	Interview format
Non-clinical cancer care specialists (9)	P1—Cancer Personalisation Lead	Group interview 1
	P2—Living with Cancer Programme Manager	
	P3—Primary Care Cancer Project Manager	
	P4—Community and Quality of Life Programme Manager	
	P5—Cancer Improvement Manager	
	P6—Cancer Programme Director	Individual interview
	P9—Community Cancer Care Coordinator	Group interview 2
	P10—Living with Cancer Community Clinical Facilitator	
	P12—Senior Cancer Care Coordinator	Individual interview
Acute clinical cancer healthcare providers (9)	P14—Cancer Information and Support Service Lead	Individual interview
	P11—Lead Nurse for Cancer, Palliative and End of Life Care	Individual interview
	P16—Respiratory Oncology Consultant	Individual interview
	P17—Lung Clinical Nurse Specialist	Group interview 3
	P18—Lung Cancer Care Co-ordinator	
	P19—Lung Clinical Nurse Specialist	Group interview 4
	P20—Lung Cancer Care Co-ordinator	
	P21—Lung Clinical Nurse Specialist	
	P22—Local Hospice Nurse Consultant	Individual interview
People with lived experience of cancer (4)	P7—Person Living with Cancer (PLWC)	Individual interview
	P8—PLWC	Individual interview
	P13—Patient and Public Involvement and Engagement (PPIE) Representative, and Informal Caregiver	Individual interview
	P15—PLWC	Individual interview

**Table 3:** Themes with indicative codes and participant quotes

Themes	Indicative codes	Indicative participant quotes
1. Fragmented communication pathways limits access	Wanting to be involved in research but unaware of it PLWC awareness of available support in rural Lincolnshire Promote services/research via different methods Disjointed community and healthcare services PLWC retell stories due to lack of information sharing Disconnect between research and clinical practice Online access challenges	‘*Patients will ask us what research is out there and we are very unsure*’ (P21 Acute clinical cancer healthcare provider)/‘*I would have quite happily taken part, but I don’t know anything*’ (P8 PLWC) ‘*All those patients in the middle of nowhere… which is down to our teams really and how we promote our service*’ (P20 Lung Cancer Care Co-ordinator) ‘*Communication in different formats… not everybody is on the internet*’ (P12 Senior Cancer Care Coordinator) ‘*Our systems don’t join up with community systems. We do the referrals and then we have no idea really what’s going on. So, it’s quite disjointed*’ (P17 Lung Clinical Nurse Specialist) ‘*[Patients] have told us about… problems with telling their story time and time again because systems just don’t talk to each other*’ (P2 Living with Cancer Programme Manager) ‘*A disconnect in terms of research and clinical practice*’ (P9 Community Cancer Care Coordinator) *‘They may not have a decent internet signal or not have a computer or not want to*’ (P7 PLWC)
2. Geographic and infrastructural disparities between urban, rural, and coastal areas	More support/opportunities in urban regions Benefits/assets to rural and coastal living Greater primary care and family support in rural areas Poor rural transport/roads affect service access and delivery More specialists in urban areas Difficult to retain staff compared to urban areas Access/travel distance barrier to/from rural/coastal areas	‘*We’ve got Lincoln, Grantham, and Boston. [Macmillan Centres] all sit in the acute hospitals’* (P10 Living with Cancer Community Clinical Facilitator) ‘*People in rural areas feel well supported… because of the tightly knit communities*’ (P2 Living with Cancer Programme Manager)/‘*Green and blue spaces*’ (P22 Local Hospice Nurse Consultant) ‘*Role of primary care… friends, family may be bigger*’ (P2 Living with Cancer Programme Manager) ‘*You haven’t got a transport infrastructure… If you get an appointment, you don’t know how on Earth you’re going to get there*’ (P13 PPIE Representative and Informal Caregiver)/‘*Carers… travel a greater distance… Bus network is rubbish… They’re going *via* cars and it takes more time, so they want to be in and out of people’s houses quicker*’ (P15 PLWC) ‘*We only have two lung oncology consultants. That is not enough [for] the amount of lung cancer patients… Our surgical patients go to Nottingham*’ (P20 Lung Cancer Care Co-ordinator) ‘*A lot of our juniors go to Nottingham, we’ve lost consultants, really good ones to Nottingham… because there’s more going on… research, the advancements*’ (P4 Clinical Nurse Specialist) ‘*Patients in Boston, Mablethorpe, Skeg[ness] have a heck of a journey… Radiotherapy is tiring enough without all that travelling*’ (P14 Cancer Information and Support Service Lead)
3. Unmet needs for living well during and after treatment	Long waiting times to deliver care to rural/coastal PLWC Need for financial and related psychological support Ask PLWC what works Employment challenges post-treatment PLWC feel lost after discharge from acute treatment Lack of post-treatment support Gap between curative and end-of-life care Need for better integration between oncology and specialist palliative care	‘*Patients have a 24-h nursing line that they can call. But again, because we’re quite rural, the response time to those calls can be quite a long time*’ (P18 Lung Cancer Care Co-ordinator) ‘*Financial concerns… Some will not put their health first… They will choose electricity bill’* (P17 Lung Clinical Nurse Specialist)/*‘Put more money in people’s pockets… reducing the psychological burden of managing finances’* (P22 Local Hospice Nurse Consultant) ‘*Asking people… what would help them access services*’ (P16 Respiratory Oncology Consultant) ‘*I can’t say [I am] reliable*’ (P15 PLWC)/‘*When patients do go back to work, people automatically assume they can do the same as… before*’ (P14 Cancer Information and Support Service Lead) ‘*They almost feel a bit lost because they’ve had the treatment… So, what happens next?*’ (P11 Lead Cancer Nurse)/‘*Re-entry back into primary care at the end… they’ve lost that support that they get from their acute team*’ (P12 Senior Cancer Care Coordinator) ‘*There’s a lot of processing… [clinicians] see the treatment as, ‘Hooray’. It’s worked… Off you go… It just doesn’t stop there*’ (P9 Community Cancer Care Coordinator)/‘*What needs to be looked at is the aftercare*’ (P8 PLWC) *‘We can’t categorise them as palliative… they’re also not curative… They sit in that in between, they’re not eligible for any of the normal support’* (P9 Community Cancer Care Coordinator) *‘If you look at NICE guidelines… oncology services should be integrated with specialist palliative care… The earlier people get specialist palliative care, they actually live longer and better… In Lincolnshire, the dosing’s wrong and it’s way too late’* (P22 Local Hospice Nurse Consultant)

*HCP*  healthcare professionals, *PLWC*   People living with cancer

### Theme 1: Fragmented communication pathways limits access

Communication barriers between healthcare providers, as well as with patients, and researchers, hindered access to information about support and research opportunities for PLWC, and professionals’ capacity to deliver equitable care, especially when multiple teams were involved. Acute professionals described a system where primary and secondary care ‘don’t really talk to each other’ (P2 Living with Cancer Programme Manager).

Professionals noted that patients had to retell their stories due to poor communication between services and, whilst many services exist, such as voluntary transport teams, PLWC were often not informed. This reflects PLWC’s accounts who shared ‘great’ community care experiences yet found ‘not a lot of people know’ about services (P15 PLWC). Whilst professionals were committed to better promoting their services, this was impeded by workforce and resource barriers:

Resources are stretched everywhere… [we are] trying to sing off the same hymn sheet, but I think from a resource perspective we know that specialist palliative care is quite challenged’ (P11 Lead Nurse for Cancer, Palliative and End of Life Care)

Some professionals explained that information about support services was delivered to patients, but at inappropriate times, such as during diagnosis, which could overwhelm patients and leave them unable to retain important information. Whilst appreciating such challenges, acute clinicians felt GP-based practitioners needed to supply information about cancer and treatments prior to diagnosis, to better prepare patients and so acting as a facilitator to their wellbeing at later stages of care:

[It] makes a massive difference to their ability to maintain their quality of life during their cancer treatment. Patients who are told by their GP that they… are likely to have a cancer are better prepared when they come and see me (P16 Respiratory Consultant)

All groups noted that information about support and the opportunity to participate in clinical research was not always suited to PLWC’s health or digital literacy, and reading levels. They identified that rural and coastal-dwelling patients ‘may either not have a decent internet signal or not have a computer or not want to’ (P7 PLWC), identifying digital inequalities that acutely affect older patients. Professionals and PLWC identified using multiple communication pathways, particularly advertisements in community venues, as facilitators to clinical research and support:

The crux of it is communication… in different formats… because not everybody is on the internet or on social media… It’s about flexibility of thinking around how you engage with people (P12 Senior Cancer Care Coordinator)

Communication deficits emerged between clinicians and researchers, as well as between clinicians and patients, which limited patients’ access to research opportunities. Whilst people with lived experience expressed a desire to participate in research, they stated professionals ‘never, never, never’ inform them about research opportunities (P13 PPIE representative and informal caregiver), as mirrored in the following account:

I would have thought maybe I might have been approached... I would have quite happily taken part, but I don’t know anything to be honest… No one’s talked about it (P8 PLWC)

These interests were not fully congruent with professionals’ views that PLWC were apprehensive about research due to unclear information and attaching ‘stigma’ to research (P9 Community Cancer Care Coordinator), which they suggested researchers should address by explaining what clinical studies involve. Whilst professionals noted information-sharing about research was improving, they felt ill-equipped to ‘know where to direct’ patients or advise on eligibility (P7 Cancer Care Coordinator). They felt professionals had differential access to research, with doctors having greater involvement, suggesting a disconnect between researchers and clinical practice which affected both groups’ access to research.

### Theme 2: Geographic and infrastructural disparities between urban, rural, and coastal areas

Study participants consistently noted a stark contrast between infrastructures in urban centres and Lincolnshire’s vast rural and coastal areas, which led to inconsistent care and ECCP implementation. Participants discussed healthcare services being situated in urban centres as creating immense travel burdens from rural and coastal areas, which were particularly challenging for patients managing symptoms and treatment side-effects:

[Travel] has an impact on quality of life… especially Lincoln having the only radiotherapy unit… patients in Boston, Mablethorpe, Skeg[ness] have a heck of a journey and radiotherapy is tiring enough without all that travelling on top (P14 Cancer Information and Support Service Lead)

Urban areas were seen to have greater staffing levels, specialised services, research innovations, and support services for PLWC compared to rural and coastal Lincolnshire. As a PPIE contributor explained, ‘there’s a total difference between urban and rural… there’s far more help’ (P13). Echoing this, a professional noted there are ‘not enough’ lung oncology consultants for the number of lung cancer patients in Lincolnshire, so ‘surgical patients go to Nottingham’ (P20 Lung Cancer Care Co-ordinator). Such challenges were exacerbated by systemic challenges with retaining care staff, leading to an overstretched workforce:

We’ve lost consultants, really good ones to Nottingham, Leicester, because there’s more going on. There’s the research… the advancements (P4 Lung Clinical Nurse Specialist)

Professionals explained that staffing crises in remote areas led patients’ families, ‘tightly knit communities’, and primary care to play ‘bigger’ roles in supporting PLWC’s wellbeing (P2 Living with Cancer Programme Manager), which exacerbated challenges for overstretched primary care systems. They characterised rural and coastal geographies as facilitators to QoL by enabling patients’ better access to ‘green [rural] and blue [coastal] spaces’ (P22 Local Hospice Nurse Consultant). However, these assets were counteracted by infrastructural barriers which limited access to care, including services located on 60 mile-an-hour roads, no motorways, poor road quality and public transport. For rural patients without a car, arranging transport came with worries that negatively affected their ‘mental health’ (P8 PLWC).

This burden of travel distance in remote areas, with hidden costs and fatigue, affected community staff who covered vast areas with limited resources, leading to long response times and compromised care, especially for out-of-hours services. PLWC empathised with these travel challenges, seen to limit the quality and consistency of care that staff could deliver:

Carers are having to travel a greater distance… The bus network is rubbish, or traveling by bike isn’t convenient, safe… [carers are] going via cars. And it takes more time, so they want to be in and out of people’s houses quicker rather than being able to do their job to a good standard (P15 PLWC)

These geographic and infrastructural disparities affected access to research opportunities, with rural and coastal Lincolnshire seen to lack hospital specialisms compared to urban and metropolitan areas, such as Nottingham, which appeared to offer ‘better access’ to research and training (P16 Respiratory Consultant). Given these challenges, which professionals noted contributed to workforce migration from rural and coastal Lincolnshire, professionals recommended offering ‘research and trials in the locality’ to facilitate research access for professionals and PLWC (P14 Cancer Information and Support Service Lead).

### Theme 3: Unmet needs for living well during and after treatment

Participants identified a critical unmet need in support for living well during and after active cancer treatment. PLWC felt abandoned after treatment, with a sudden drop-off in support for re-adjusting to life that they saw as a priority area to improve. This directly compromised patients’ wellbeing in survivorship, as one PLWC reflected:

Aftercare and supportive care… wasn’t as good as the care when you’re actually going through and having all your treatments... like, oh, you’ve finished your treatment. That’s it. So, I think there is a gap (P8 PLWC)

Professionals across roles had similar experiences of patients feeling ‘lost’ after treatment (P11 Lead Cancer Nurse), particularly at the point of re-entering primary care when ‘they’ve lost that support that they get from their acute team’ (P12 Senior Cancer Care Coordinator). Professionals noted a mismatch between service pathways and support that ends upon successful treatment, and PLWC’s ongoing need for support in processing their experiences through survivorship:

There’s a lot of processing… [clinicians] see the treatment as, ‘Hooray’. It’s worked… Go and live your life… It just doesn’t stop there (P9 Community Cancer Care Coordinator)

Professionals identified a GP postcode lottery: the level and quality of post-treatment support depended on where patients live; and gaps in care were greater when GPs relied on signposting to community services that face volunteer shortages. Some patients fell in a gap where services could not ‘categorise them as palliative’ or ‘curative’ (P9 Community Cancer Care Coordinator), leaving them in a state of crisis and requiring emergency care. Specialist palliative care professionals noted gaps in integrating with oncology services, contradicting ECCP and National Institute for Health and Care Excellence guidance and limiting timely access to specialist support:

We don’t have integrated specialist palliative care services into cancer services. We just don’t have the input. We [Palliative care services] should be [involved] much earlier on. We could improve people’s outcomes (P22 Local Hospice Nurse Consultant)

PLWC reported unmet needs for managing the knock-on effects of treatment on their work life and financial security; one PLWC (P8) stated they ‘didn’t get any income for a year’. Yet, professionals noted that these challenges were often not understood by employers and colleagues who ‘assume people can do the same as… before’ (P14 Cancer Information and Support Service Lead). PLWC struggled to sustain employment as they ‘can’t say [I am] reliable’ (P5). Professionals noted that financial challenges hindered PLWC from accessing post-treatment support, with trade-offs between health and paying ‘the electricity bill’ (P17 Lung Clinical Nurse Specialist), especially for rural and coastal PLWC on temporary seasonal contracts:

We had a patient last summer due to have surgery just as she was about to start her annual seasonal work. She was really hesitant… because she was going to miss out on her annual seasonal work, because that is the only work she got (P6 Clinical Nurse Specialist)

**Fig. 2:**
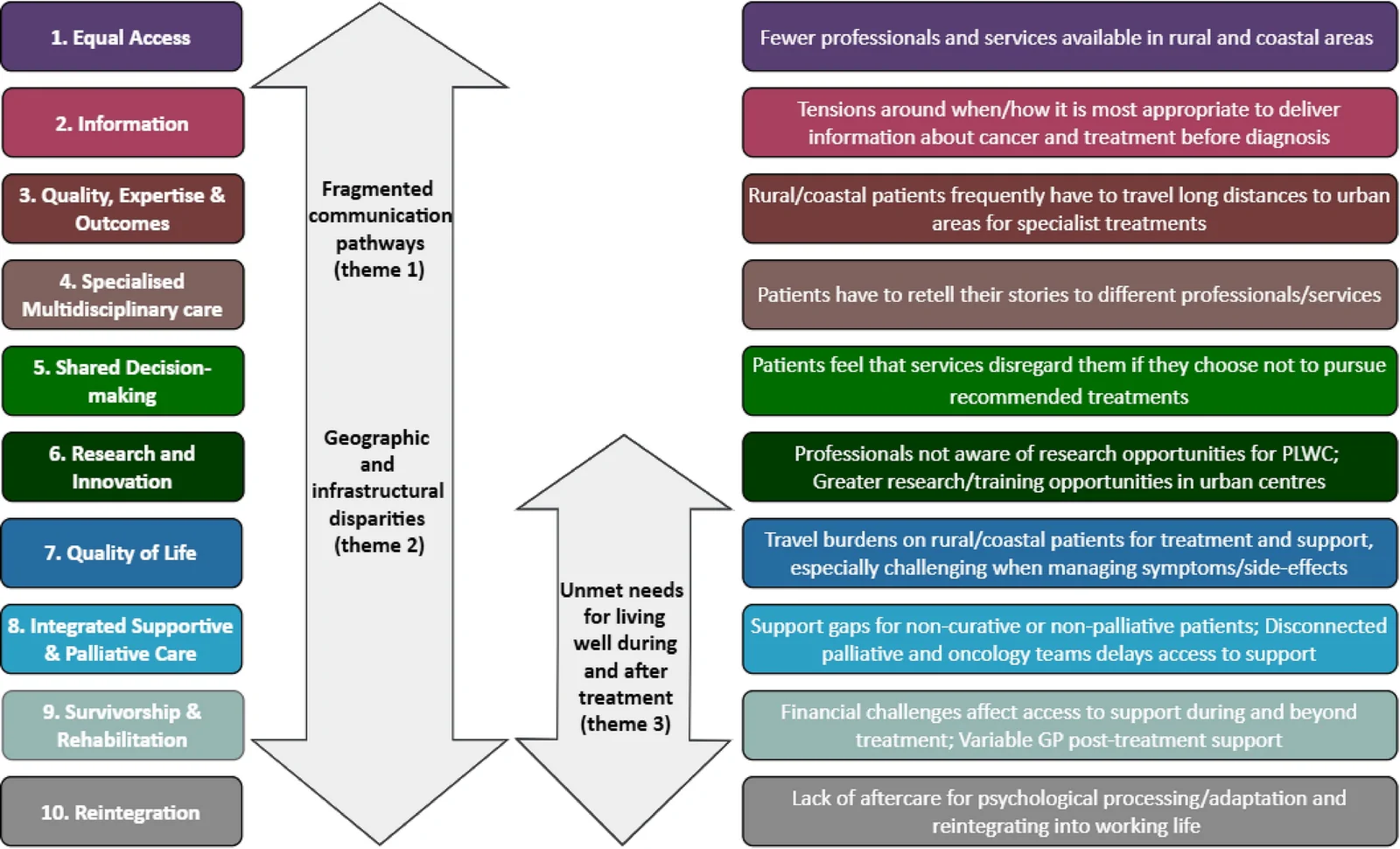
Participant-reported barriers to implementing the ECCP in rural and coastal Lincolnshire

Such dilemmas between health and finances for patients and their families limited access to post-treatment support, breaching the ECCP. PLWC who decided not to pursue specific treatments reported that services did not offer alternative support, leaving them feeling disregarded and characterising this as: ‘oh well, you’re beyond this’ (P15 PLWC). Professionals recommended ‘asking [patients] what would help them access services’ (P16 Respiratory Oncology Consultant) to embed a patient-centred approach in care as a facilitator to ECCP implementation. Overall, the findings indicate stark communication, workforce and infrastructural challenges, and opportunities, for ECCP implementation in rural and coastal settings, as summarised in Fig. 2.

## Discussion

Given the challenges with ECCP implementation in other European [12] and rural contexts [5, 11], this study examined the barriers and facilitators to implementing the ECCP in a UK-based rural and coastal county, according to cancer care professionals, and people with lived experience. It describes the novel use of the ECCP policy framework as a research tool to examine implementation, providing a template for researchers; and illuminates the value of qualitative methods for identifying place-specific, contextual complexities that affect ECCP implementation [28]. Overall, workforce and infrastructure challenges, created travel and communication barriers to support and research, for rural and coastal individuals, therefore breaching the ECCP.

Communication deficits hindered professionals from fulfilling the specialised multidisciplinary and integrated care ECCP rights across services, as they had to routinely ask patients for information discussed with other providers [29]. Poor information-sharing about patient needs can compromise the continuity and quality of cancer care [30–32]. Research highlights gaps in integration between specialist palliative care and oncology services, as reported by our rural and coastal participants, which risks delaying access to palliative care that could improve longevity and QoL [29]. Communication deficits were greater in rural and coastal contexts, due to resource challenges that limit contact between urban specialists and localised primary care providers [23, 33, 34]. Our findings thus echo challenges in rural settings in high-income countries, including Australia, Canada, and the USA [16, 35]. Scholarly inquiry into coastal cancer care and outcomes is a relatively new phenomenon unique to the UK [7, 23], though recent work identifies global similarities and a need to recognise the unique needs of coastal settings beyond the UK [21].

Rural and coastal patients negotiated immense travel distances to access routine and specialist care, often without viable transport options, creating barriers to support and affecting patients’ QoL and rights to be involved in clinical research. Increased travel distances to healthcare services correlate with poorer cancer survival outcomes [6, 14]. Rural patients often receive delayed diagnoses, shorter durations of chemotherapy, and decline treatment [36]. Whilst researchers posit digital solutions to travel burdens as a facilitator to support [37], digital exclusion was a barrier to rural and coastal patients’ ECCP ‘information’ rights, linked to age-related and internet connectivity challenges [38, 39]. Efforts to strengthen digital care capabilities [40] must avoid further excluding these groups and include in-person information about support and research in community venues, such as village shops, and the use of community champions [11] to facilitate more equitable access.

Participants in this study, and elsewhere [41, 42], note barriers to attracting and retaining medical, nursing, and allied health professionals in rural and coastal settings, leading to shortages and an overstretched workforces, compromising integrated care. The National Cancer Plan for England aims to increase trainee positions in these settings, including specialist cancer nurses, and is a welcome development which may increase capacity for communication and help address shortages [43]. As workforce challenges hindered professionals’ contact with research institutions, akin to other resource-limited rural contexts [34], it is unsurprising that PLWC in this study reported not receiving information about research from care providers, despite being open to participating. International evidence suggests that rural PLWC are often unaware of clinical trials available to them [44, 45], so they may miss out on research that could improve their outcomes [46], thus impeding their QoL and research and innovation rights. It is vital to strengthen communication pathways to equitably implement the ECCP and the National Cancer Plan for England aims to promote access to trials for potentially life-saving treatments, regardless of where people live [43].

Despite these widely discussed infrastructural challenges [5, 11, 47], our study identifies rural and coastal assets, including close-knit social networks, blue and green spaces, as potential facilitators to wellbeing, as noted elsewhere [48, 49] and a priority for future research [11]. Service providers could leverage these assets to implement the ECCP; for example, running support activities in, or near, sea water, which could support survivors’ psychological healing [50]. This could help address gaps in psychological support from primary care, particularly prominent for PLWC who were not eligible for curative or end-of-life care, as found previously [32]. Whilst the ECCP sets out patients’ right to discuss their needs with their care teams [13], they were not always supported after declining treatment, compromising the principle of person-centred care. It is vital to address aftercare gaps for rural and coastal PLWC and enhance professional awareness of, and capacity for, person-centred discussions to facilitate ECCP implementation.

PLWC faced gaps in support for their financial security and reintegrating into work. The effects of cancer and associated treatments often leave survivors to need multimodal financial, psychosocial, and physical support [11] as well as employer flexibility [51]. Our study shows that PLWC may minimise, or decline, treatment and support that clashes with temporary seasonal work in seaside tourism and agricultural industries, adding contextual nuance to evidence of the costs of living with cancer [52, 53]. Echoing review findings [51], workplace colleagues placed unrealistic expectations on PLWC’s performance during and after treatment. Whilst the National Cancer Plan aims to increase support for returning to work [43], it is unclear how this considers rural and coastal PLWC’s unique travel and employment situations, as well as how to improve workplace awareness and adjustments for their particular cancer-related challenges. It is necessary to address these gaps to ensure PLWC’s QoL beyond formal care settings. The findings indicate recommendations for policy, healthcare organisations, professionals, and researchers, outlined in Table 4.

**Table 4:** Recommendations for Policy, Services, Professionals, and Researchers to Facilitate ECCP Implementation

Domain	Recommendation
Policy	Promoting cross-national learning of good practice to identify effective approaches for implementing the ECCP in diverse rural and coastal contexts
	ECCP implementation must be supported with dedicated resources and guidance rather than relying solely on policy endorsement
	Taking a multi-sectorial and integrated approach to ECCP implementation, particularly to improve workplace awareness about supporting PLWC
Healthcare organisations	Integrate the ECCP into local cancer care pathway, design implementation strategies that recognise the challenges of rural and coastal areas. For example, access, workforce shortages, travel, and access to specialist care
	Utilise multiple (digital and in-person, community-based) methods to communicate cancer research opportunities and support services to improve access for rural and coastal PLWC
	Establish processes to monitor and evaluate implementation, including Patient-Reported Experience Measures and Outcome Measures
Healthcare professionals	More outreach to improve awareness and understanding of the ECCP with cancer professionals, particularly around person-centred treatment discussions and strategies to promote PLWC’s inclusion in research
	Embed the ECCP principles into routine clinical interactions and shared decision-making to work with rural and coastal patients and communities to better understand barriers to implementation and solutions
Researchers	Build stronger and flexible partnerships with cancer care professionals in rural and coastal settings, to support information-sharing about research opportunities with, and for, diverse professional groups and PLWC
	Further research on rural and coastal cancer care, especially the benefits of green and blue spaces, to inform bespoke interventions, in line with prior research [11]

### Limitations

There are several limitations. First, participants were recruited through established networks with cancer professionals and PLWC, which could bias the sample to more homogeneous perspectives. Regardless, it should be noted that our sample included a wide range of cancer professionals who support PLWC across primary and secondary care, and in the community.

Second, the study included four PLWC, compared to eighteen professionals, limiting access to PLWC’s viewpoints. However, much of the existing qualitative evidence predominately focuses on patient perspectives and as such our study makes a valuable contribution to the rural and coastal cancer literature from an explicit professional and workforce perspective. Limited demographic data is reported about professionals to ensure their anonymity in this narrow, geographic context, which could limit transferability.

Third, whilst patients did not directly feed into the study design and interpretation of findings, the ECCP framework that informed the study design, data collection and analysis, is a patient-centred statement that has been extensively co-produced with PLWC, patient advocates, and professionals. Finally, with regards to PLWC’s sociodemographic backgrounds, they were in remission from cancer, so their experiences may not reflect those recently diagnosed or undergoing active treatment. Similar to the professional participants, detailed demographic data were not collected as the study solely focused on their experiences of receiving cancer care. However, as experiences may vary depending on cancer type, treatment plans, prognoses, and socio-demographic identities, such as ethnicity [54], future research could examine stratified and tumour-specific differences. Online data collection also limited access to this study for digitally excluded participants.

## Conclusion

This study reports in detail the perspectives of cancer professionals, and people with lived experience of cancer regarding barriers and facilitators to implementing the ECCP in rural, coastal, and urban Lincolnshire. It identifies critical gaps in communication and information-sharing between healthcare providers, and with research institutions and patients. Challenges in implementing the ECCP domains is exacerbated by workforce barriers. The right to QoL was unrealised due to significant travel burdens (immense distances and limited public and personal transport options) to care and support. Community-based professionals also had to cover vast distances to deliver care, with shortages in staff and resources, leading to long waiting times and inconsistencies, therefore hindering patients’ from having equal access to quality care, information, support, and research opportunities.

Workforce capacity challenges hindered integration of specialist palliative care into oncology services in rural and coastal contexts; a barrier that must be addressed to ensure better long-term patient outcomes and thus fulfil patients’ ECCP rights to integrated and supportive palliative care. The findings of this study suggest a need to improve post-treatment care, which actively considers rural and coastal patients’ support needs around financial management, returning to work (especially in seasonal roles), psychological wellbeing, as well as digital exclusion. Leveraging rural and coastal assets, such as green and blue spaces and local support networks, as part of an enhanced offer of post-treatment support could improve patients’ QoL, and overall ECCP rights. The new National Cancer Plan for England commits to enhancing cancer care in rural and coastal settings [43]. This commitment should be supported as a matter of urgency to address the disparities identified in this study.

## Supplementary Information

Below is the link to the electronic supplementary material.

Supplementary file1 (DOCX 24 KB)

Supplementary file2 (DOCX 25 KB)

## Data Availability

The anonymised data (transcripts) used in this manuscript are available upon reasonable request by contacting the corresponding author. Each request will be considered on a case-by-case basis. The dataset has not been deposited in a public repository due to the ethical approval as well as the increased sensitivity of this qualitative dataset, which focuses on individual perspectives in a specific geographic region.

## References

[1] European Cancer Organisation (2014) European Code of Cancer Practice. https://www.europeancancer.org/content/european-code-of-cancer-practice.html

[2] Phillips JL, Currow DC (2010) Cancer as a chronic disease. Collegian 17:47–50. 10.1016/j.colegn.2010.04.007 20738055

[3] Smith SR, Zheng JY, Silver J et al (2020) Cancer rehabilitation as an essential component of quality care and survivorship from an international perspective. Disabil Rehabil 42:8–13. 10.1080/09638288.2018.1514662 30574818

[4] Leach CR, Weaver KE, Aziz NM et al (2015) The complex health profile of long-term cancer survivors: prevalence and predictors of comorbid conditions. J Cancer Surviv 9:239–251. 10.1007/s11764-014-0403-1 25319681

[5] McPeake K, Jeanes L, Nelson D et al (2023) Developing a ‘living with cancer’ programme in a rural and coastal setting: experiences of collaborative and innovative co-production across an integrated health system. J Cancer Policy 38:100452. 10.1016/j.jcpo.2023.100452 37931888

[6] Afshar N, English DR, Milne RL (2019) Rural–urban residence and cancer survival in high-income countries: a systematic review. Cancer 125:2172–2184. 10.1002/cncr.32073 30933318

[7] Nelson D, Calanzani N, Pickwell-Smith B et al (2025) Invisible geographies - the rural and coastal blind spot in UK cancer policy: a content analysis. J Cancer Policy 46:100650. 10.1016/j.jcpo.2025.100650 41101659

[8] Han L, Sullivan R, Tree A et al (2024) The impact of transportation mode, socioeconomic deprivation and rurality on travel times to radiotherapy and surgical services for patients with prostate cancer: a national population-based evaluation. Radiother Oncol 192:110092. 10.1016/j.radonc.2024.110092 38219910

[9] Lawler M, Oliver K, Gijssels S et al (2021) The European code of cancer practice. J Cancer Policy 28:100282. 10.1016/j.jcpo.2021.100282 35559911

[10] Lawler M, Le Chevalier T, Murphy MJ et al (2014) A catalyst for change: the European cancer patient’s bill of rights. Oncologist 19:217–224. 10.1634/theoncologist.2013-0452 24493667 PMC3958470

[11] Nelson D, Selby P, Kane R et al (2024) Implementing the European code of cancer practice in rural settings. J Cancer Policy 39:100465. 10.1016/j.jcpo.2023.100465 38184144

[12] Kufel-Grabowska J, Jassem J (2026) Prospects for implementing the European code of cancer practice: lessons from Poland. Health Policy 166:105581. 10.1016/j.healthpol.2026.105581 41707561

[13] Lawler M, Price R, Gijssels S et al (2021) Implementing the European code of cancer practice to improve Europe’s cancer outcomes. Oncol Hematol 17:9. 10.17925/OHR.2021.17.1.9

[14] Carriere R, Adam R, Fielding S et al (2018) Rural dwellers are less likely to survive cancer – an international review and meta-analysis. Health Place 53:219–227. 10.1016/j.healthplace.2018.08.010 30193178

[15] Nelson D, Lawler M, Kane R et al (2025) A national cancer plan for England: remember the needs of people in rural and coastal areas. J Cancer Policy 43:100553. 10.1016/j.jcpo.2024.100553 39716618

[16] Bhatia S, Landier W, Paskett ED et al (2022) Rural–urban disparities in cancer outcomes: opportunities for future research. JNCI J Natl Cancer Inst 114:940–952. 10.1093/jnci/djac030 35148389 PMC9275775

[17] Scott ECS, Hoskin PJ (2024) Health inequalities in cancer care: a literature review of pathways to diagnosis in the United Kingdom. eClinicalMedicine 76:102864. 10.1016/j.eclinm.2024.102864 39398494 PMC11470173

[18] Cu A, Meister S, Lefebvre B, Ridde V (2021) Assessing healthcare access using the Levesque’s conceptual framework– a scoping review. Int J Equity Health 20:116. 10.1186/s12939-021-01416-3 33962627 PMC8103766

[19] Kenny A, Dickson-Swift V, Carlin A et al (2026) Oral health interventions to improve access in rural areas of high-income countries: a mixed methods systematic review. Comm Dent Oral Epidemiol 54:273–284. 10.1111/cdoe.70058 PMC13146141 41708571

[20] Schwandt TA (1994) Constructivist, interpretivist approaches to human inquiry. SAGE, London, pp 118–137

[21] Nelson D, Pickwell-Smith B, Falvey J et al (2026) Coastal cancer care: a blueprint to address the unmet needs through place-based research and policy approaches. J Cancer Policy 49:100769. 10.1016/j.jcpo.2026.100769 42372821

[22] Office for National Statistics (2026) 2021 Rural Urban Classification. https://www.ons.gov.uk/methodology/geography/geographicalproducts/ruralurbanclassifications/2021ruralurbanclassification

[23] Department of Health and Social Care (2021) Chief Medical Officer’s annual report 2021: health in coastal communities. https://www.gov.uk/government/publications/chief-medical-officers-annual-report-2021-health-in-coastal-communities

[24] Braun V, Clarke V (2022) Thematic analysis: a practical guide. SAGE Publications

[25] Byrne D (2022) A worked example of Braun and Clarke’s approach to reflexive thematic analysis. Qual Quant 56:1391–1412. 10.1007/s11135-021-01182-y

[26] Tong A, Sainsbury P, Craig J (2007) Consolidated criteria for reporting qualitative research (COREQ): a 32-item checklist for interviews and focus groups. Int J Qual Health Care 19:349–357. 10.1093/intqhc/mzm042 17872937

[27] Braun V, Clarke V (2021) One size fits all? What counts as quality practice in (reflexive) thematic analysis? Qual Res Psychol 18:328–352. 10.1080/14780887.2020.1769238

[28] Barnes M, Matka E, Sullivan H (2003) Evidence, understanding and complexity: evaluation in non-linear systems. Evaluation 9:265–284. 10.1177/13563890030093003

[29] Wang C, LeBlanc TW (2025) Palliative care integration in oncology: a review and update. J Hosp Palliat Care 28:121–130. 10.14475/jhpc.2025.28.4.121 41368512 PMC12683178

[30] Harrison R, Newman B, Chauhan A et al (2026) Care my way: co-designing a patient-held resource to improve information sharing between primary and specialist care for people with cancer. Health Expect 29:e70498. 10.1111/hex.70498 41486562 PMC12765981

[31] Alessy SA, Alhajji M, Rawlinson J et al (2022) Factors influencing cancer patients’ experiences of care in the USA, United Kingdom, and Canada: a systematic review. eClinicalMedicine 47:101405. 10.1016/j.eclinm.2022.101405 35497061 PMC9046116

[32] Collaço N, Lippiett KA, Wright D et al (2024) Barriers and facilitators to integrated cancer care between primary and secondary care: a scoping review. Support Care Cancer 32:120. 10.1007/s00520-023-08278-1 38252169 PMC10803398

[33] Ratnapradipa KL, Ranta J, Napit K et al (2022) Qualitative analysis of cancer care experiences among rural cancer survivors and caregivers. J Rural Health 38:876–885. 10.1111/jrh.12665 35381622 PMC9492624

[34] Kumar V, Martinez-Martin N, Olson NW (2026) Ethical issues in rural health research: a scoping review. Bioeth Inq 23:181–192. 10.1007/s11673-025-10453-4 40788451

[35] Ugalde A, Jongebloed H, Wright C et al (2026) Towards equitable cancer outcomes for rural and remote communities: reflections, lessons and recommendations. Lancet Reg Health West Pac 66:101756. 10.1016/j.lanwpc.2025.101756 41647886 PMC12869289

[36] Levit LA, Byatt L, Lyss AP et al (2020) Closing the rural cancer care gap: three institutional approaches. JCO Oncol Pract 16:422–430. 10.1200/OP.20.00174 32574128

[37] Morris BB, Rossi B, Fuemmeler B (2022) The role of digital health technology in rural cancer care delivery: a systematic review. J Rural Health 38:493–511. 10.1111/jrh.12619 34480506 PMC8894502

[38] Asthana S, Prime S (2023) The role of digital transformation in addressing health inequalities in coastal communities: barriers and enablers. Front Health Serv 3:1225757. 10.3389/frhs.2023.1225757 37711604 PMC10498291

[39] Age UK (2021) Ageing in coastal and rural communities. https://www.ageuk.org.uk/our-impact/policy-research/ageing-in-coastal-and-rural-communities/

[40] European Commission (2024) Europe’s Beating Cancer Plan: Making a difference. https://commission.europa.eu/topics/public-health/european-health-union/cancer-plan-europe_en

[41] Gillespie J, Cosgrave C, Malatzky C, Carden C (2022) Sense of place, place attachment, and belonging-in-place in empirical research: a scoping review for rural health workforce research. Health Place 74:102756. 10.1016/j.healthplace.2022.102756 35168027

[42] Scheil-Adlung X (2015) Global evidence on inequities in rural health protection. New data on rural deficits in health coverage for 174 countries. International Labour Organization

[43] Department of Health and Social Care and NHS England (2026) National Cancer Plan for England. https://www.gov.uk/government/publications/national-cancer-plan-for-england

[44] Geana M, Erba J, Krebill H et al (2017) Searching for cures: inner-city and rural patients’ awareness and perceptions of cancer clinical trials. Contemp Clin Trials Commun 5:72–79. 10.1016/j.conctc.2016.12.004 29740623 PMC5936702

[45] Jacobson I, Markley P, Kamboj J (2026) Rural–urban differences in cancer worry, beliefs, and attitudes towards research progress. Cancer Causes Control 37:64. 10.1007/s10552-026-02147-y 41854765

[46] Boaz A, Hanney S, Jones T, Soper B (2015) Does the engagement of clinicians and organisations in research improve healthcare performance: a three-stage review. BMJ Open 5:e009415. 10.1136/bmjopen-2015-009415 26656023 PMC4680006

[47] Nelson D, McGonagle I, Jackson C et al (2022) A rural‐urban comparison of self‐management in people living with cancer following primary treatment: a mixed methods study. Psychooncology 31:1660–1670. 10.1002/pon.6011 35971265 PMC9804546

[48] Graham F, Kane R, Gussy M, Nelson D (2022) Recovery of health and wellbeing in rural cancer survivors following primary treatment: analysis of UK qualitative interview data. Nurs Rep 12:482–497. 10.3390/nursrep12030046 35894036 PMC9326683

[49] Van Der Kruk SR, Butow P, Mesters I et al (2022) Psychosocial well-being and supportive care needs of cancer patients and survivors living in rural or regional areas: a systematic review from 2010 to 2021. Support Care Cancer 30:1021–1064. 10.1007/s00520-021-06440-1 34392413 PMC8364415

[50] Britton E, Kindermann G, Domegan C, Carlin C (2020) Blue care: a systematic review of blue space interventions for health and wellbeing. Health Promot Int 35:50–69. 10.1093/heapro/day103 30561661 PMC7245048

[51] Greidanus MA, De Boer AGEM, De Rijk AE et al (2018) Perceived employer‐related barriers and facilitators for work participation of cancer survivors: a systematic review of employers’ and survivors’ perspectives. Psychooncology 27:725–733. 10.1002/pon.4514 28753741

[52] Haier J, Schaefers J (2022) Economic perspective of cancer care and its consequences for vulnerable groups. Cancers (Basel) 14:3158. 10.3390/cancers14133158 35804928 PMC9265013

[53] Bourgeois A, Horrill T, Mollison A et al (2024) Barriers to cancer treatment for people experiencing socioeconomic disadvantage in high-income countries: a scoping review. BMC Health Serv Res 24:670. 10.1186/s12913-024-11129-2 38807237 PMC11134650

[54] Kelly-Brown J, Palmer Kelly E, Obeng-Gyasi S et al (2022) Intersectionality in cancer care: a systematic review of current research and future directions. Psychooncology 31:705–716. 10.1002/pon.5890 35199401

