# Supplementary material for: Barriers and facilitators to implementing the European code of cancer practice in a rural and coastal setting: a qualitative study
Source: Cancer Causes Control. 2026 Aug 8;37(9):140. doi: 10.1007/s10552-026-02225-1 (PMC13452836; doi:10.1007/s10552-026-02225-1)
Supplement: Supplementary file 1 [file 10552_2026_2225_MOESM1_ESM.docx]

**Supplementary Information 1:**

**Title**: Barriers and Facilitators to Implementing the European Code of Cancer Practice in a Rural and Coastal Setting: A Qualitative Study

**Journal**: Cancer Causes & Control

**Authors**: Shanice Thomas, Hannah Cumberland Keeble, Erica Brown, Sarah R Baker, Samuel Cooke, Mark Lawler, Kathie McPeake, Kathy Oliver, Peter Selby, David Nelson

**Corresponding Author**: David Nelson, Lincoln Institute for Rural and Coastal Health, University of Lincoln, Lincoln, UK. [Dnelson@lincoln.ac.uk](mailto:Dnelson@lincoln.ac.uk)

**Interview Topic Guide for Professionals and PLWC**

Part 1: Researcher re-shares the ECCP infographic (Figure 1) and explains the research aim.

Part 2: Participant background:

- Professionals: Please introduce yourself and give some background to your professional role in Lincolnshire and how you have been involved with cancer care?

Probes could include: type and location of service; length of time in current role

- People with lived experience: Please introduce yourself and give some background to how you have been involved with cancer care, such as having personal experience?

Probes could include: rural/coastal location; type of cancer services accessed

Part 3: Participant experiences and challenges with ECCP:

- Example question: What have been your experiences of accessing/delivering cancer care in Lincolnshire, and any challenges you have faced?
- Probe to what extent these experiences relate to ECoCP domains, for example: ‘Equal access’; ‘information’ about disease and treatment; information about the ‘quality, expertise & outcomes’ of care; ‘specialised multidisciplinary care’; and ‘shared decision-making’
- Research and Innovation – example questions and probes:
- To what extent are PLWC/professionals able to engage with and access cancer research in Lincolnshire?
- What are some of the challenges with regards to cancer research in Lincolnshire?
- How might we improve access to and engagement with cancer research in Lincolnshire?

Probes could include: access to research, lack of engagement with research, awareness of research, lack of opportunities for research, “research literacy”, training, lack of research infrastructure, rural-coastal-urban, clinical versus non-clinical research.

- Quality-of-life (QoL) – example questions and probes:
- What things can impact on PLWC’s quality of life in Lincolnshire?
- How might we improve quality of life for PLWC in Lincolnshire?

Probes: physical QoL, emotional QoL, practical QoL, cancer-specific QoL, does QoL differ across the county (e.g. coastal-urban-rural), QoL based on levels of affluence/deprivation/health literacy.

- Integrated supportive and palliative care – example questions and probes:
- To what extent do PLWC in Lincolnshire receive optimal supportive and palliative care?
- What are some of the challenges with delivering integrated supportive and palliative care in Lincolnshire?
- How might we improve supportive and palliative care in Lincolnshire?

Probes could include: access, distance to care, time implications with travel, informal caregivers, isolation as disease progresses, rural-urban-coastal, workforce and resource implications.

- Survivorship and rehabilitation – example questions and probes:
- To what extend do PLWC in Lincolnshire have opportunities to receive and discuss with their care team a clear, managed and achievable plan for their survivorship and rehabilitation?
- What are some of the challenges with survivorship and rehabilitation for PLWC in Lincolnshire?
- How might we improve survivorship and rehabilitation for PLWC in Lincolnshire?

Probes could include: relationship with primary care/GPs, dynamics between primary-acute-community care, survivorship, and rehabilitation needs? Differences across the county between rural-urban-coastal, role of close friends/family.

- Reintegration – example questions and probes:
- To what extent do PLWC in Lincolnshire face challenges with reintegrating into society following their diagnosis/treatment?
- How might we improve how PLWC in Lincolnshire reintegrate into society following their diagnosis/treatment?

Probes could include returning to work, socialising, issues of stigma and discrimination, ‘place-based’ depending on rural-urban-coastal locations.

Part 4: Final reflections: Is there anything we have not discussed about implementing the ECoCP, that you would like to add about your experiences of cancer care in Lincolnshire?
