# Supplementary material for: Barriers and facilitators to implementing the European code of cancer practice in a rural and coastal setting: a qualitative study
Source: Cancer Causes Control. 2026 Aug 8;37(9):140. doi: 10.1007/s10552-026-02225-1 (PMC13452836; doi:10.1007/s10552-026-02225-1)
Supplement: Supplementary file 2 [file 10552_2026_2225_MOESM2_ESM.docx]

**Supplementary Information 2:**

**Title**: Barriers and Facilitators to Implementing the European Code of Cancer Practice in a Rural and Coastal Setting: A Qualitative Study

**Journal**: Cancer Causes & Control

**Authors**: Shanice Thomas, Hannah Cumberland Keeble, Erica Brown, Sarah R Baker, Samuel Cooke, Mark Lawler, Kathie McPeake, Kathy Oliver, Peter Selby, David Nelson

**Corresponding Author**: David Nelson, Lincoln Institute for Rural and Coastal Health, University of Lincoln, Lincoln, UK. [Dnelson@lincoln.ac.uk](mailto:Dnelson@lincoln.ac.uk)

**Consolidated Criteria for Reporting Qualitative Studies (COREQ): 32-item checklist**, **based on Tong et al (2007)**

| No. | Topic/Item | Description | Page number |
| --- | --- | --- | --- |
| **Domain 1: Research team and reflexivity** | | |  |
| Personal characteristics | | |  |
| *1.* | Interviewer/facilitator | Which author/s conducted the interview or focus group? | 6 |
| *2.* | Credentials | What were the researcher's credentials? *E.g. PhD, MD* | 1, 6 |
| *3.* | Occupation | What was their occupation at the time of the study? | 6 |
| *4.* | Gender | Was the researcher male or female? | 6 |
| *5.* | Experience and training | What experience or training did the researcher have? | 6 |
| Relationship with participants | | |  |
| *6.* | Relationship established | Was a relationship established prior to study commencement? | 6 |
| *7.* | Participant knowledge of the interviewer | What did the participants know about the researcher? *E.g. Personal goals, reasons for doing the research* | 6-7 |
| *8.* | Interviewer characteristics | What characteristics were reported about the interviewer/facilitator? *E.g. Bias, assumptions, reasons and interests in the research topic* | 6-7 |
| **Domain 2: Study design** | | |  |
| Theoretical framework | | |  |
| *9.* | Methodological orientation and theory | What methodological orientation was stated to underpin the study? *E.g. grounded theory, discourse analysis, ethnography, phenomenology, content analysis* | 6 |
| Participant selection | | |  |
| *10.* | Sampling | How were participants selected? *E.g. purposive, convenience, consecutive, snowball* | 6 |
| *11.* | Method of approach | How were participants approached? *E.g. face-to-face, telephone, mail, email* | 7-8 |
| *12.* | Sample size | How many participants were in the study? | N/A |
| *13.* | Non-participation | How many people refused to participate or dropped out? What were the reasons for this? | N/A |
| Setting | | |  |
| *14.* | Setting of data collection | Where was the data collected? *E.g. home, clinic, workplace* | 6 |
| *15.* | Presence of nonparticipants | Was anyone else present besides the participants and researchers? | 6 |
| *16.* | Description of sample | What are the important characteristics of the sample? *E.g. demographic data, date* | 7-8 |
| Data collection | | | |
| *17.* | Interview guide | Were questions, prompts, guides provided by the authors? Was it pilot tested? | 6, see supplementary file |
| *18.* | Repeat interviews | Were repeat interviews carried out? If yes, how many? | N/A |
| *19.* | Audio/visual recording | Did the research use audio or visual recording to collect the data? | 6 |
| *20.* | Field notes | Were field notes made during and/or after the interview or focus group? | N/A |
| *21.* | Duration | What was the duration of the interviews or focus group? | 6 |
| *22.* | Data saturation | Was data saturation discussed? | N/A – not congruent with a constructivist design |
| *23.* | Transcripts returned | Were transcripts returned to participants for comment and/or correction? | N/A |
| **Domain 3: Analysis and findings** | | | |
| Data analysis | | | |
| *24.* | Number of data coders | How many data coders coded the data? | 7 |
| *25.* | Description of the coding tree | Did authors provide a description of the coding tree? | 9-10 |
| *26.* | Derivation of themes | Were themes identified in advance or derived from the data? | 6-7 |
| *27.* | Software | What software, if applicable, was used to manage the data? | 7 |
| *28.* | Participant checking | Did participants provide feedback on the findings? | N/A |
| Reporting | | | |
| *29.* | Quotations presented | Were participant quotations presented to illustrate the themes / findings? Was each quotation identified? *E.g. Participant number* | 9-15 |
| *30.* | Data and findings consistent | Was there consistency between the data presented and the findings? | 9-16 |
| *31.* | Clarity of major themes | Were major themes clearly presented in the findings? | 9-16 |
| *32.* | Clarity of minor themes | Is there a description of diverse cases or discussion of minor themes? | 9-15 |
